# Hepatopancreas transcriptome analyses provide new insights into the molecular regulatory mechanism of fast ovary maturation in *Macrobrachium nipponense*

**DOI:** 10.1186/s12864-022-08851-8

**Published:** 2022-08-31

**Authors:** Sufei Jiang, Wenyi Zhang, Yiwei Xiong, Dan Cheng, Jisheng Wang, Shubo Jin, Yongsheng Gong, Yan Wu, Hui Qiao, Hongtuo Fu

**Affiliations:** 1 Key Laboratory of Freshwater Fisheries and Germplasm Resources Utilization, Ministry of Agriculture and Rural Affairs, Freshwater Fisheries Research Center, Chinese Academy of Fishery Sciences, Wuxi, Jiangsu People’s Republic of China; 2 Wuxi Fisheries College, Nanjing Agricultural University, Wuxi, Jiangsu People’s Republic of China

**Keywords:** *Macrobrachium nipponense*, Transcriptome analysis, Hepatopancreas, Reproduction

## Abstract

**Background:**

*Macrobrachium nipponense* is an economically and ecologically important freshwater prawn that is widely farmed in China. In contrast to other species of marine shrimp, *M. nipponense* has a short sexual maturity period, resulting in not only high stocking densities, but also a reduced survival rate and increased risk of hypoxia. Therefore, there is an urgent need to study the molecular mechanisms underlying fast ovary maturation in this species*.*

**Results:**

Comparative transcriptome analysis was performed using hepatopancreatic tissue from female *M. nipponense* across five ovarian maturation stages to explore differentially expressed genes and pathways involved in ovarian maturation. In total, 118.01 Gb of data were generated from 15 transcriptomes. Approximately 90.46% of clean reads were mapped from the *M. nipponense* reference genome. A comprehensive comparative analysis between successive ovarian maturation stages generated 230–5814 differentially expressed genes. Gene Ontology (GO) enrichment was highly concentrated in the “biological process” category in all four comparison groups, and mainly focused on energy synthesis and accumulation, energy decomposition and transport. Kyoto Encyclopedia of Genes and Genomes (KEGG) enrichment results showed that, among 20 significantly enriched KEGG pathways, nine were involved in the synthesis, degradation, and metabolism of carbohydrates, lipids, and other nutrient intermediates, suggesting that the hepatopancreas has an important role in energy supply during ovarian maturation. Furthermore, the “Insect hormone biosynthesis” pathway was found to have a dominant role in the development of the ovary from immaturity to maturity, supporting the hypothesis that ecdysteroid- and juvenile hormone-signaling pathways have an important role in hepatopancreas regulation of ovarian maturation.

**Conclusion:**

Taken together, this study sheds light on the role of the hepatopancreas in the molecular regulation of ovary maturation in *M. nipponense*. The present study provided new insights for understanding the mechanisms of reproductive regulation in crustaceans.

## Introduction

*Macrobrachium nipponense*, commonly known as the oriental river prawn, is widely distributed throughout China, except in Tibet and Qinghai province [1]. It is an economically important and popular aquacultural species because of its low susceptibility to disease and its delicious taste, with production exceeding 240,000 tons in China in 2020 [2]. The Yangtze River basin is the main area *M. nipponense* aquaculture, with Jiangsu province accounting for half of the *M. nipponense* aquaculture output in China.

The short sexual maturity period of female *M. nipponense* leads to rapid mass self-reproduction in autumn [3]. During the breeding season, ovarian maturation of *M. nipponense* accelerates with increases in water temperature. By early autumn, newborn females develop to sexual maturity and lay eggs, only 45 days after hatching. This autumn reproduction produces numerous offspring, which results in the presence of multiple generations of prawns in aquaculture ponds. This can lead to reduced resilience and survival rates and increased risks of hypoxia [4]. The number of large prawns also decreases significantly during autumn reproduction, which seriously affected the economic benefits of prawn breeding. Therefore, it is of relevance to analyze the mechanisms involved in fast sexual maturation to provide insights that could help address these issues.

The ovary is an important tissue in crustacean sexual maturity. Molecular studies of female *M. nipponense* ovary have primarily focused on transcriptome analyses of ovarian development stages [5–7]. Many genes, such as those encoding vitellogenin, vitellogenin receptor, cyclin A, opsin, gonad-inhibiting hormone, ribosomal protein L24, and cathepsin L, have been identified and demonstrated to have important roles in ovary maturation [8–14]. In addition to the ovaries, the hepatopancreas is also reported to have an important role in ovarian maturation, supplying not only energy, but also essential fatty acids and cholesterol for the synthesis of sex steroid hormones [15]. Furthermore, the hepatopancreas is an important site for the synthesis of vitellogenin. In several crustacean species, such as *Carcinus maenas*, *Eriocheir sinensis*, *Marsupenaeus japonicas*, *Penaeus monodon*, *M. nipponense*, and *Scylla paramamosain*, the hepatopancreas makes a significantly higher contribution to vitellogenin synthesis compared with the ovary [11, 16–19]. In previous studies, ovary transcriptomes of five developmental stages were constructed to detect the key pathways and genes involved in ovary maturation [6]. Many differentially expressed genes (DEGs) and enriched Kyoto Encyclopedia of Genes and Genomes (KEGG) pathways were obtained after a series of comparative transcriptome analyses [9, 13, 14]. Based on these results, it was concluded that expression patterns of many genes involved in ovarian maturation, including those encoding vitellogenin and three cathepsin L family genes, were significantly higher in hepatopancreas than in ovary. Therefore, we hypothesized that the hepatopancreas in crustaceans had an important role in regulating ovarian maturation. However, the molecular mechanisms involved in the function of the hepatopancreas in ovarian maturation are unclear.

In this study, we used high-throughput sequencing to obtain hepatopancreas transcriptomes of female *M. nipponense* during five ovarian maturation stages. DEGs between stages were identified and enriched to reveal the genes or pathways involved in the regulation of ovarian maturation. These data provided new insights for understanding the mechanisms of reproductive regulation in crustaceans. They also provided a theoretical basis for solving many of the issues facing *M. nipponense* aquaculture that result from fast sexual maturation in this species.

## Material and methods

### Sample preparation

Healthy adult female *M. nipponense* at different stages of ovarian maturation [*N* = 100, body weight (BW) ± SD: 2.24 ± 0.52 g] for transcriptome analysis were obtained from Dapu Scientific Experimental Base, Freshwater Fisheries Research Center (Wuxi, China). All prawns were kept in a recirculating aquarium system for 1 week. Ovarian staging was defined according to a previous study [12]. The ovaries were assigned to maturation classes by color: OI (transparent, undeveloped stage), OII (yellow, developing stage), OIII (light green, nearly-ripe stage), OIV (dark green, ripe stage), and OV (gray, spent stage). Eighteen prawns were randomly selected from each of the five ovarian maturation stages, with three repetitions per stage. The hepatopancreas from each prawn at each ovarian maturation stages was dissected into liquid nitrogen and stored at − 80 °C for further study.

### The cDNA library construction and sequencing

Hepatopancreatic tissues from 15 samples from each stage were homogenized with RNAiso Plus Reagent (TaKaRa, Japan) to extract total RNA. The frozen samples were quickly transferred to a mortar precooled with liquid nitrogen and ground to powder. Then, additional RNAiso Plus was added and the mix ground again. The homogenate was transferred into a centrifuge tube and centrifuged at 12,000 ≥ *g* at 4 °C for 5 min. The supernatant was transferred into a new centrifuge tube and purified with chloroform and isopropyl alcohol. The resulting supernatant was transferred into a new centrifuge tube and centrifuged with 12,000×*g* at 4 °C for 10 min. The precipitate was dried and dissolved with RNA-free water. The purity and concentration of RNA were detected by a Nanodrop 2000 spectrophotometer (Thermo Fisher Scientific, Waltham, MA, USA). The RNA integrity was evaluated by an Agilent 2100 (Agilent Technologies, Santa Clara, CA, USA).

Three μg RNA per sample was used to construct the cDNA library by using a NEBNext Ultra RNA Library Prep Kit for Illumina (NEB, Ipswich, MA, USA) according to the manufacturer”s protocol. The RNA was purified and broken into small random fragments using poly-T oligo-attached magnetic beads (Life Technologies, Carlsbad, CA, USA). The first-strand cDNA was synthesized with random hexamers using mRNA as template and the second-strand cDNA was then synthesized by adding buffer, dNTPs, RNase H, and DNA polymerase I. The cDNA fragments in the library with a length of 150–200 bp were screened and purified using an Ampure XP system (Beckman Coulter, Beverly, MA, USA). The purified double-stranded cDNA was then repaired at the end, a-tailed, and sequenced. AMPure XP Beads were used for fragment size selection. Finally, a cDNA library was obtained by PCR enrichment. Qubit 2.0 was used for preliminary quantifications. Agilent 2100 was used to detect the library insert size. The prepared libraries were then sequenced on an Illumina HiSeq X Ten by Genepioneer Biotechnologies Company (Nanjing, China).

### RNA-seq analysis

Raw data were filtered to obtain high quality clean data by removing adapters and low quality reads. The generated cleaned reads were then aligned to the *M. nipponense* reference genome (NCBI Accession No.: ASM1510439v1, https://ftp.cngb.org/pub/CNSA/data2/CNP0001186/CNS0254395/CNA0014632/) using Tophat2 to obtain the mapped data. The aligned reads from each sample were assembled by Stringtie (http://ccb.jhu.edu/software/stringtie). The expression levels of genes were calculated by reads per kilobase per million mapped reads (RPKM) [20]. Differential expression analysis, functional annotation, and functional enrichment were performed according to the gene expression levels in different samples. DEGs were determined by DESeq (www.bioconductor.org/packages/release/bioc/html/DESeq2.html), using a fold-change (FC) ≥2 and false discovery rate (FDR) < 0.05 as screening criteria.

### GO and KEGG enrichment analysis of DEGs

Gene ontology (GO) enrichment (www.geeontology.org) and KEGG enrichment (https://www.kegg.jp/kegg/kegg1.html) were performed to find the GO terms and KEGG pathways that were significantly enriched in HE-I versus HE-II, HE-II versus HE-III, HE-III versus HE-IV, and HE-IV versus HE-V compared with the whole genome (E-value ≤10^− 5^).

### Validation of RNA-Seq and further investigation of candidate genes

Nine DEGs were selected from the significantly enriched KEGG pathways to verify the RNA-seq results by qPCR. We also analyzed the expression profiles of these nine genes in five ovarian maturation stages. RNA isolation from tissue and cDNA synthesis were performed using an RNAiso Plus kit and a reverse transcriptase M-MLV kit (TaKaRa) according to the manufacturer’s protocols. The *EIF* gene of *M. nipponense* was used as an internal control gene [21] and the qPCR reaction system and procedures were consistent with our previous study [22]. Expression levels were calculated by the 2^− ΔΔCT^ method [23] and the data were analyzed using one-way ANOVA and two-tailed Student’s *t*-test in SPSS 23.0. Data are presented as the mean ± standard deviation and differences were significant at *P* < 0.05. The amino acid sequences of two candidate genes were used to generate the phylogenetic trees with MEGA5.0 based on the neighbor joining (NJ) method and bootstrapping replications were 1000.

## Results

### Overview of hepatopancreas transcriptomes in different ovarian maturation stages

In total, 15 hepatopancreas transcriptomes were constructed for sequencing, generating 118.01 Gb of data. The clean reads of each sample were no less than 6.74 Gb of data. The average clean reads and clean data were 393,382,795 and 118,014,838,500, respectively; each set of sample data are detailed in Table 1. The average Q20 and Q30 percentage content was 97.54 and 93.21%, respectively. The average GC content of each sample was 41.40% (Table 2). Approximately 85.41–92.57% of clean reads mapped to the reference genome of *M. nipponense*, with an average mapping rate of 90.46%*.*

**Table 1:** Sequencing data statistics of 15 hepatopancreas transcriptomes

Sample	Clean reads	Clean data (bp)	GC (%)	Q20 (%)	Q30 (%)	Mapped reads	Mapping ratio (%)
HE-I-1	24,978,523	7,493,556,900	42.67	97.27	92.59	44,412,158	88.90
HE-I-2	31,115,877	9,334,763,100	42.17	97.61	93.28	56,461,687	90.73
HE-I-3	23,763,113	7,128,933,900	40.43	97.37	92.75	42,457,924	89.34
HE-II-1	26,105,848	7,831,754,400	42.28	97.80	93.70	47,776,850	91.51
HE-II-2	22,463,945	6,739,183,500	40.83	97.83	93.75	40,983,499	91.22
HE-II-3	30,265,569	9,079,670,700	41.54	97.72	93.44	55,379,753	91.49
HE-III-1	25,465,241	7,639,572,300	41.62	97.78	93.61	46,945,225	92.18
HE-III-2	25,596,125	7,678,837,500	41.46	97.79	93.69	46,573,437	90.98
HE-III-3	24,360,454	7,308,136,200	43.81	97.88	94.00	44,798,107	91.95
HE-IV-1	27,951,273	8,385,381,900	43.89	97.74	93.58	51,525,222	92.17
HE-IV-2	25,887,156	7,766,146,800	43.77	97.77	93.66	47,927,371	92.57
HE-IV-3	23,058,462	6,917,538,600	42.75	97.73	93.60	42,271,786	91.66
HE-V-1	24,816,743	7,445,022,900	36.07	96.93	92.09	44,339,091	89.33
HE-V-2	30,601,957	9,180,587,100	39.49	97.04	92.36	53,538,358	87.48
HE-V-3	26,952,509	8,085,752,700	38.15	96.80	92.01	46,039,085	85.41
total/average	393,382,795	118,014,838,500	41.40	97.54	93.21	711,429,553	90.46

**Table 2:** Summary of significantly enriched GO terms

Comparison group	Term_ID	Term description	Gene number	q-value
HE-I vs. HE-II	GO:0045329	carnitine biosynthetic process	6	0.00010017
	GO:0006101	citrate metabolic process	4	0.00017043
	GO:0006633	fatty acid biosynthetic process	8	0.00017197
	GO:0006085	acetyl-CoA biosynthetic process	4	0.0005792
	GO:0006334	nucleosome assembly	6	0.00152825
	GO:2001295	malonyl-CoA biosynthetic process	3	0.00247781
	GO:0005975	carbohydrate metabolic process	10	0.00213486
	GO:0030258	lipid modification	3	0.01559932
	GO:0006807	nitrogen compound metabolic process	3	0.03237204
HE-II vs. HE-III	GO:0048477	oogenesis	12	0.00156306
	GO:0006633	fatty acid biosynthetic process	5	0.00120518
	GO:0016042	lipid catabolic process	4	0.02989402
	GO:0005975	carbohydrate metabolic process	5	0.04897362
HE-III vs. HE-IV	GO:0002011	morphogenesis of an epithelial sheet	3	0.0030563
	GO:0006635	fatty acid beta-oxidation	5	0.01525708
	GO:0072089	stem cell proliferation	4	0.02496115
	GO:0006857	oligopeptide transport	3	0.04034019
	GO:0019439	aromatic compound catabolic process	2	0.03890067
HE-IV vs. HE-V	GO:0006412	translation	58	4.45E-09
	GO:0000398	mRNA splicing, via spliceosome	69	1.53E-05
	GO:0015992	proton transmembrane transport	16	0.00052087
	GO:0032543	mitochondrial translation	21	0.00528481
	GO:0051301	cell division	71	0.0047298
	GO:0006364	rRNA processing	33	0.00563453
	GO:0015986	ATP synthesis coupled proton transport	11	0.00516878
	GO:0040035	hermaphrodite genitalia development	18	0.00848067
	GO:0071902	positive regulation of protein serine/threonine kinase activity	8	0.01193306
	GO:0006633	fatty acid biosynthetic process	19	0.01214723
	GO:0006754	ATP biosynthetic process	9	0.01548962
	GO:0008406	gonad development	34	0.02115767
	GO:0006383	transcription by RNA polymerase III	7	0.02786654
	GO:0042273	ribosomal large subunit biogenesis	7	0.0263184
	GO:0006085	acetyl-CoA biosynthetic process	6	0.03171148

### DEG identification

**Fig. 1:**
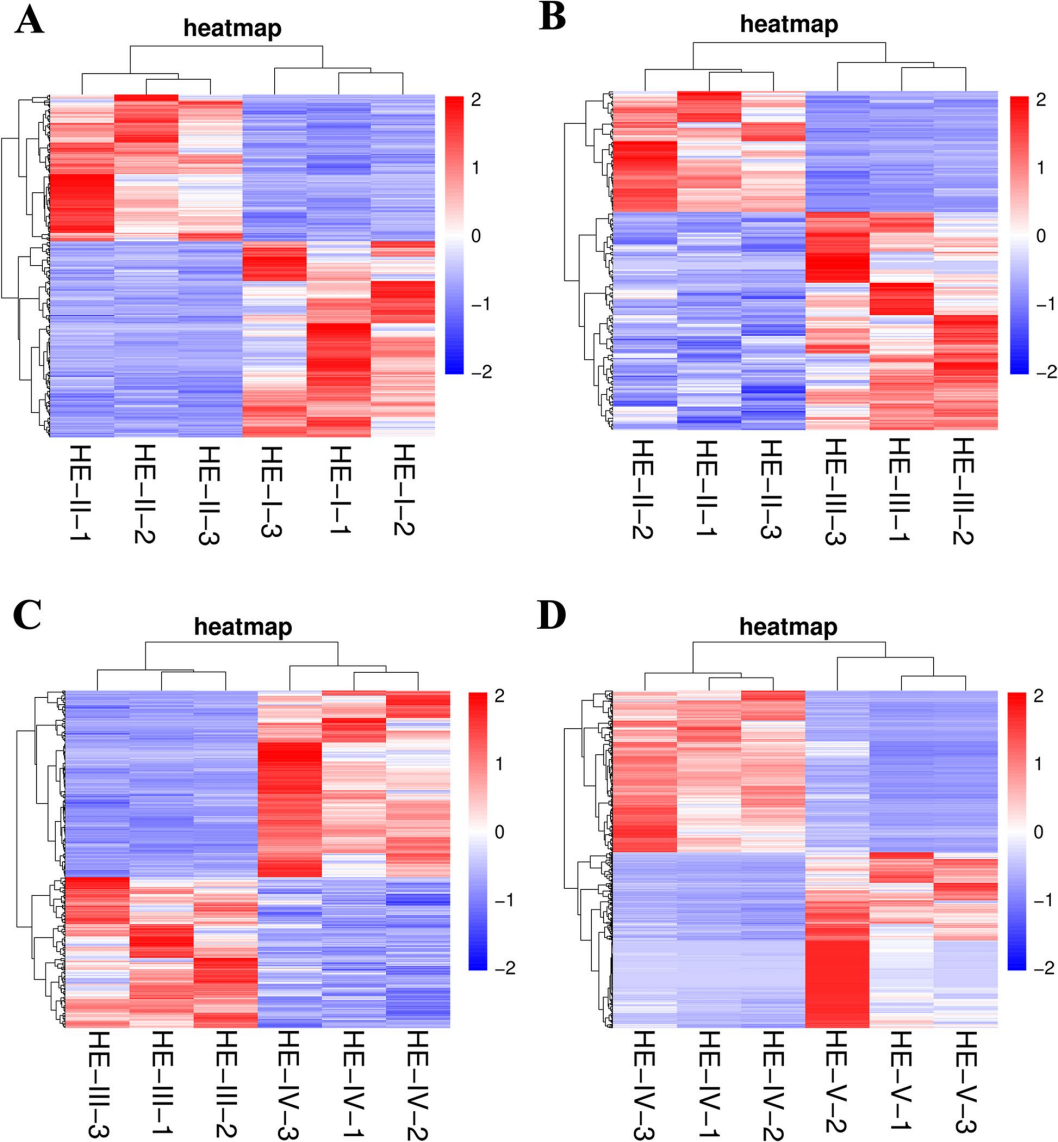
Heatmap of a total six samples in different comparison groups. **A** HE-I vs. HE-II, **B** HE-II vs. HE-III, **C** HE-III vs. HE-IV, **D** HE-IV vs. HE-V. The expression level of DEGs descended as color changed from deep red to deep blue based on FPKM value

Comparative transcriptome analyses in HE-I versus HE-II, HE-II versus HE-III, HE-III versus HE-IVIV and HE-IV versus HE-V were performed to identify DEGs involved in ovarian maturation (log_2_ ratio ≥ 1, *P* < 0.05). In HE-I versus HE-II, 535 DEGs were identified, with 229 upregulated and 306 downregulated. In HE-II versus HE-III and HE-III versus HE-IV, there were 230 (148 upregulated and 82 downregulated) and 481 DEGs (266 upregulated and 215 downregulated), respectively. The number of DEGs detected in HE-IV versus HE-V (5814) was nearly 10 times that in other comparisons, with 3028 upregulated and 2786 downregulated. The results of the DEG heat map analysis in HE-I versus HE-II, HE-II versus HE-III, HE-III versus HE-IV and HE-IV versus HE-V are shown in Fig. 1. All these results indicated that the expression pattern of unigenes in four comparison groups were significantly different, especially in HE-IV versus HE-V.

### GO enrichment of DEGs

**Fig. 2:**
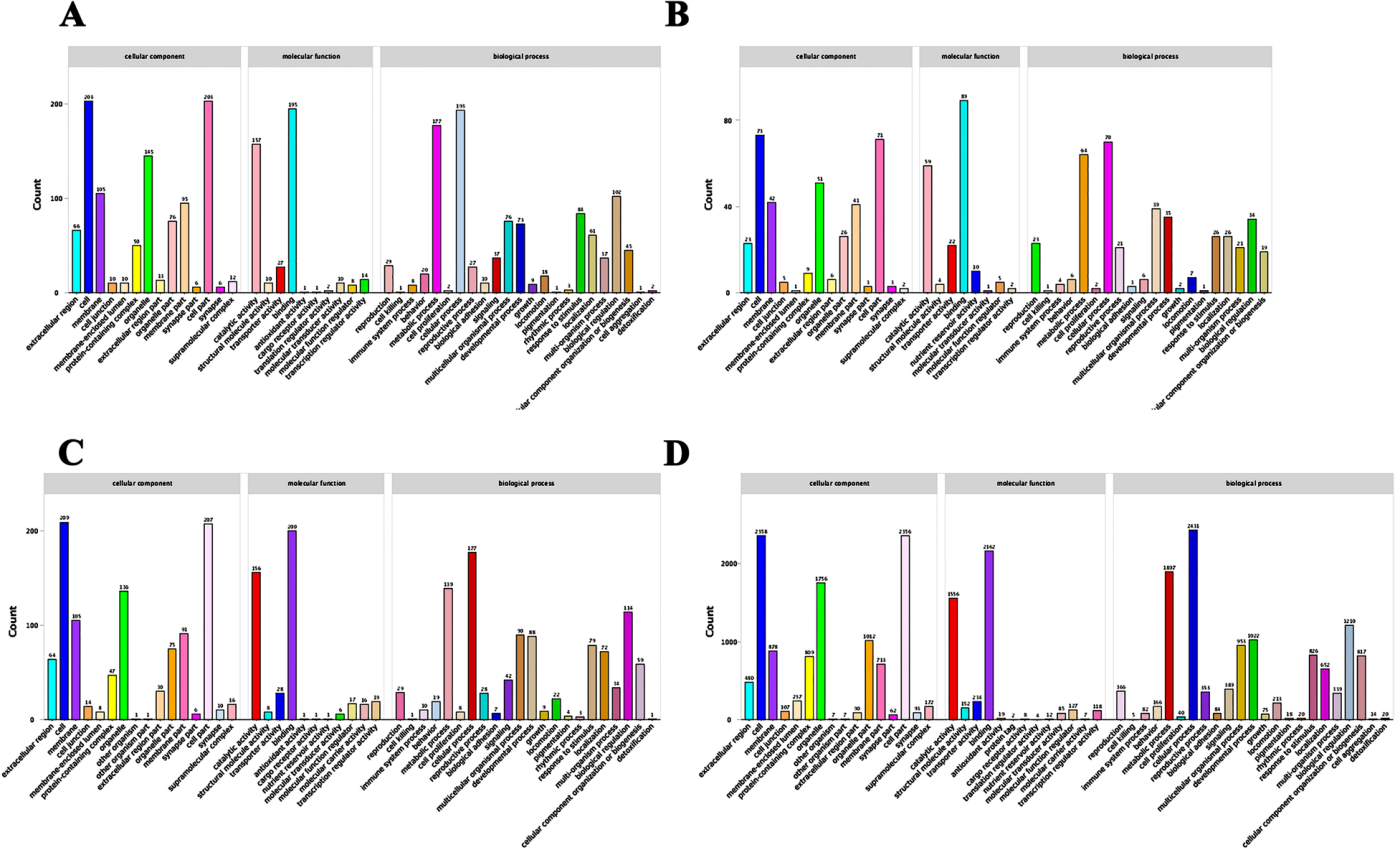
GO enrichment analysis of DEGs in different comparison groups. **A** HE-I vs. HE-II, **B** HE-II vs. HE-III, C: HE-III vs. HE-IV, D: HE-IV vs. HE-V

For GO enrichment (Fig. 2), all DEGs in each comparison group were categorized into three groups: molecular functions, cellular components, and biological processes. In HE-I versus HE-II, there were 14, 10, and 23 GO terms in these groups, respectively, among which the highest classification terms were “cell” and “cell part” (203 DEGs). In HE-II versus HE-III, the enriched number of GO terms was 14, eight, and 20, respectively, and “binding” was the most-represented classification term (89 DEGs). In HE-III versus HE-IV, there were 16, 11, and 22 enriched GO terms in the three categories, respectively, and the “cell” terms in the “cellular component” category was the highest classification terms (209 DEGs). In HE-IV versus HE-V, there were 16, 13, and 23 enriched terms, respectively, and “cellular process” terms in “biological process”were the highest classification terms (2431 DEGs). In all four comparison groups, the “biological process” category had the most GO terms (Table 2; *q*-value < 0.05).

### KEGG pathway enrichment of DEGs

**Fig. 3:**
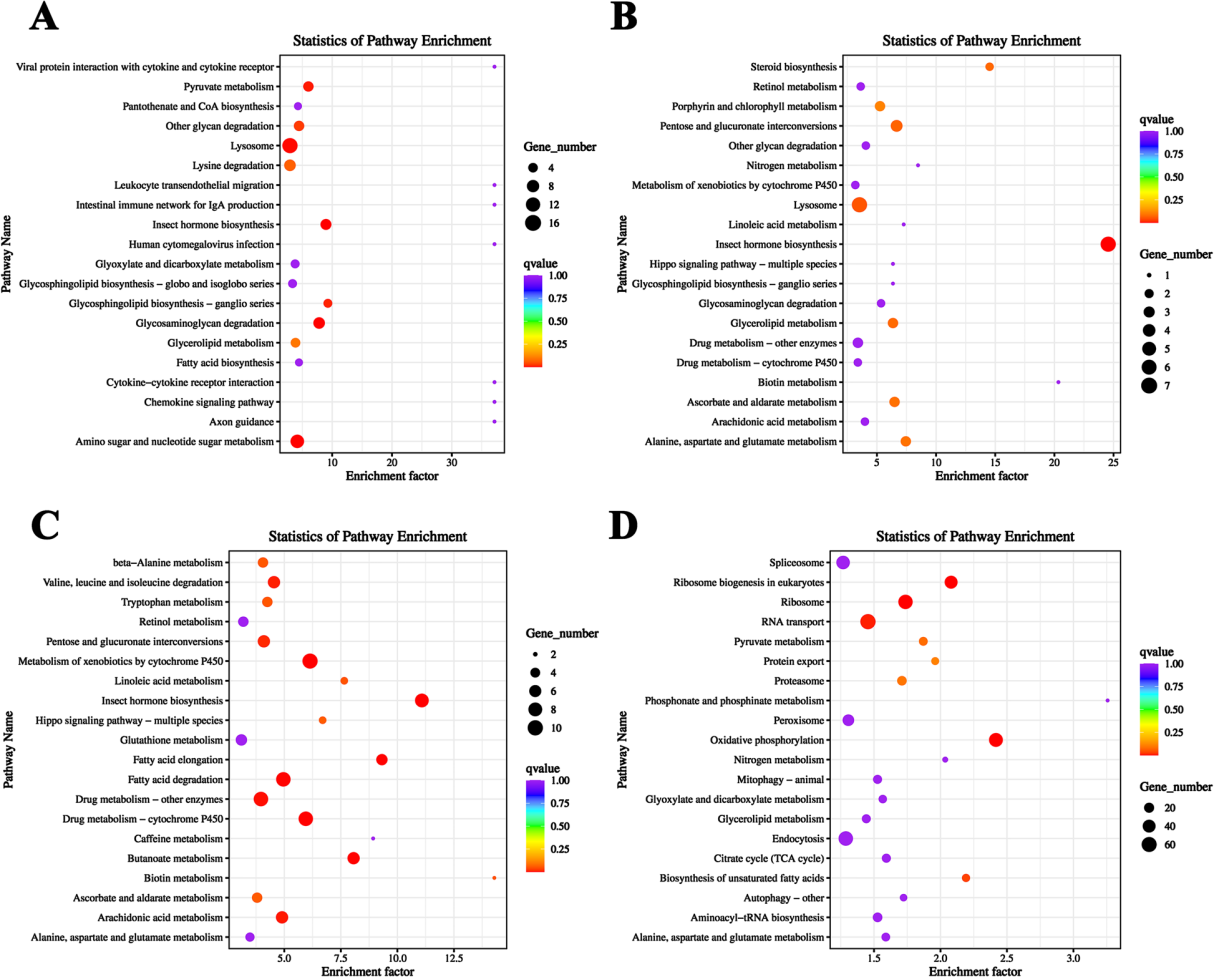
KEGG pathway enrichment analysis of DEGs in different comparison groups. **A** HE-I vs. HE-II, **B**: HE-II vs. HE-III, **C** HE-III vs. HE-IV, **D** HE-IV vs. HE-V

All the DEGs in each comparison group were also analyzed for KEGG pathway enrichment. In HE-I versus HE-II, 126 DEGs were enriched into 95 KEGG pathways. In HE-II versus HE-III, there were 49 DEGs grouped into 48 KEGG pathways. In HE-III versus HE-IV, 131 DEGs were enriched into 98 KEGG pathways, whereas in, HE-IV versus HE-V, the DEG number was 1149 and the KEGG pathway number was 137. The top 20 significantly enriched KEGG pathways in each comparison group are showed in Fig. 3. The top 20 most significantly enriched KEGG pathways (*q*-value < 0.05) in each comparison group are summarized in Table 3.

**Table 3:** Summary of enriched significantly KEGG pathways

Comparison group	Pathways	Pathway ID	Gene number	q-value
HE-I vs. HE-II	Glycosaminoglycan degradation	map00531	8	0.0005225
	Insect hormone biosynthesis	map00981	7	0.000394
	Amino sugar and nucleotide sugar metabolism	map00520	12	0.0007705
	Lysosome	map04142	16	0.0020308
	Pyruvate metabolism	map00620	7	0.0075439
	Glycosphingolipid biosynthesis - ganglio series	map00604	4	0.011246
	Other glycan degradation	map00511	6	0.02767
HE-II vs. HE-III	Insect hormone biosynthesis	map00981	7	3.52E-07
HE-III vs. HE-IV	Insect hormone biosynthesis	map00981	9	4.91E-06
	Metabolism of xenobiotics by cytochrome P450	map00980	11	5.61E-05
	Drug metabolism - cytochrome P450	map00982	10	0.00015748
	Butanoate metabolism	map00650	7	0.00042295
	Fatty acid degradation	map00071	10	0.00050501
	Fatty acid elongation	map00062	6	0.00047923
	Drug metabolism - other enzymes	map00983	10	0.01395453
HE-IV vs. HE-V	Oxidative phosphorylation	map00190	57	3.23E-11
	Ribosome biogenesis in eukaryotes	map03008	47	1.90E-06
	Ribosome	map03010	61	4.97E-05
	RNA transport	map03013	70	0.00819226
	Biosynthesis of unsaturated fatty acids	map01040	14	0.03374109

### Transcriptome validation of RNA-seq with RT-qPCR

**Fig. 4:**
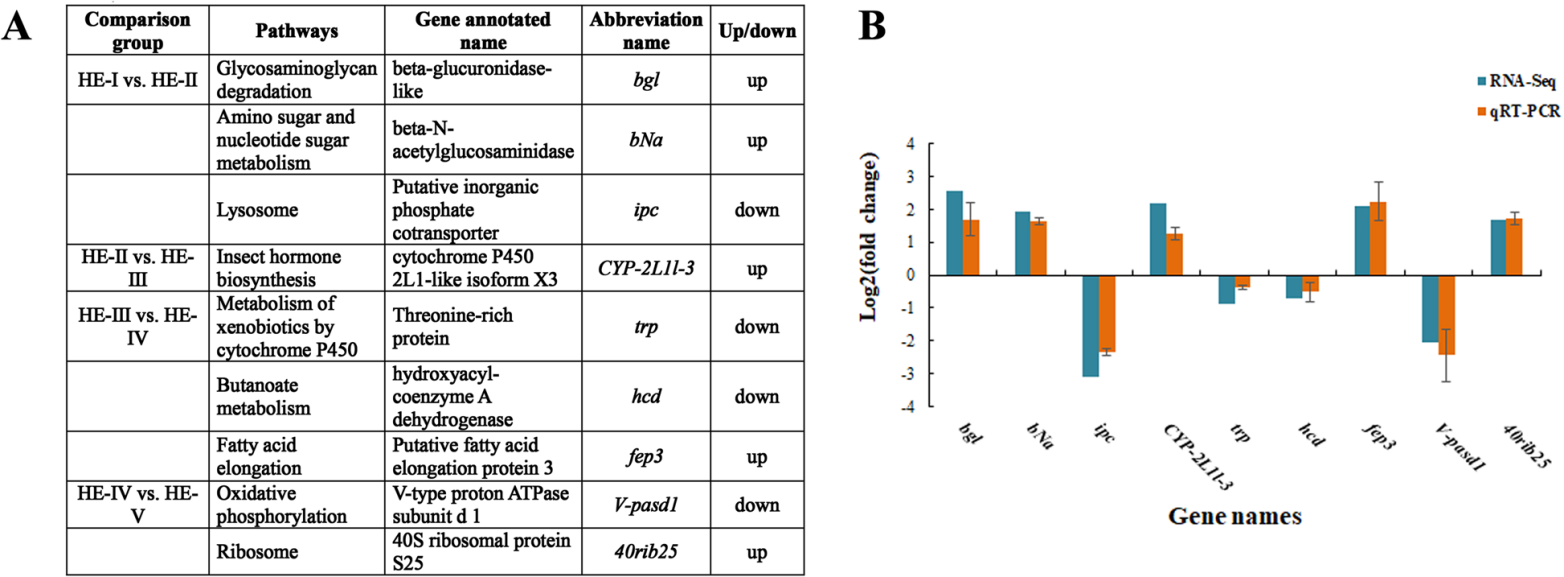
qPCR validation of RNA-Seq. **A** Summarized information of 9 selected DEGs. **B** Expression comparison of the selected genes by RNA-Seq and qPCR. The fold changes by RNA-Seq and qPCR were calculated by FPKM and 2^− ΔΔCT^, respectively. qPCR data are shown as means ± SD (*n* = 3)

To validate the gene expression level by RNA-seq, nine genes were randomly selected from 20 significantly enriched KEGG pathways for qPCR validation. The nine genes (*bgl*, *bNa*, *ipc*, *CYP-2L1l-3*, *trp*, *hcd*, *fep3*, *V-pasd1*, and *40rib25*) are detailed in Fig. 4A. Primers were designed by Primer 5.0 and are listed in Table 4. The qPCR results showed consistent results with those from transcriptome expression data (Fig. 4B), which confirmed the accuracy by transcriptome analysis (*P* < 0.05).

**Table 4:** List of primers used for qPCR

Gene name	Abbreviation name	Forward and reverse primer (5′ -3′)
beta-glucuronidase-like	*bgl*	CACTTGGAAATCATTGAGCAGCA TTCAGCCTGGTATTCTTCTGTCC
beta-N-acetylglucosaminidase	*bNa*	AAGACTCTCCGGTGTTTCCTTAC GTAGACAGGGAAAGAGTGTGTGT
Putative inorganic phosphate cotransporter	*ipc*	GGGGAATCAAACAATCACAGCTT TCTGTTTGGGTTTTGGTTCGTTC
cytochrome P450 2 L1-like isoform X3	*CYP-2L1l-3*	CATTCTCTTCCTCTCCCTCGTTC AGGGCATCCTTCATCAGTTTGTA
Threonine-rich protein	*trp*	AGGAGGAATGTTGAAAGCCGTAT CGGAATCAGACGAAGAAAAGCAC
hydroxyacyl-coenzyme A dehydrogenase	*hcd*	CAGATGCAATACAACGATTGGGT TGCATTCAACTGTGACTTTTCCC
Putative fatty acid elongation protein 3	*fep3*	CGAGAAACGACGATGGATGAAAG CATGATTGCCCAGGACATAGAGA
V-type proton ATPase subunit d 1	*V-pasd1*	TTGAAGGAGCTGGAAACAATCCT TGTCAATCTTTGCCCTGTGTTTC
40S ribosomal protein S25	*40rib25*	CCCAAGAAGGATACCAAGGGAAA TCCGGTGATCTTGAGTCTTTCAG

### Further investigation of candidate genes

**Fig. 5:**
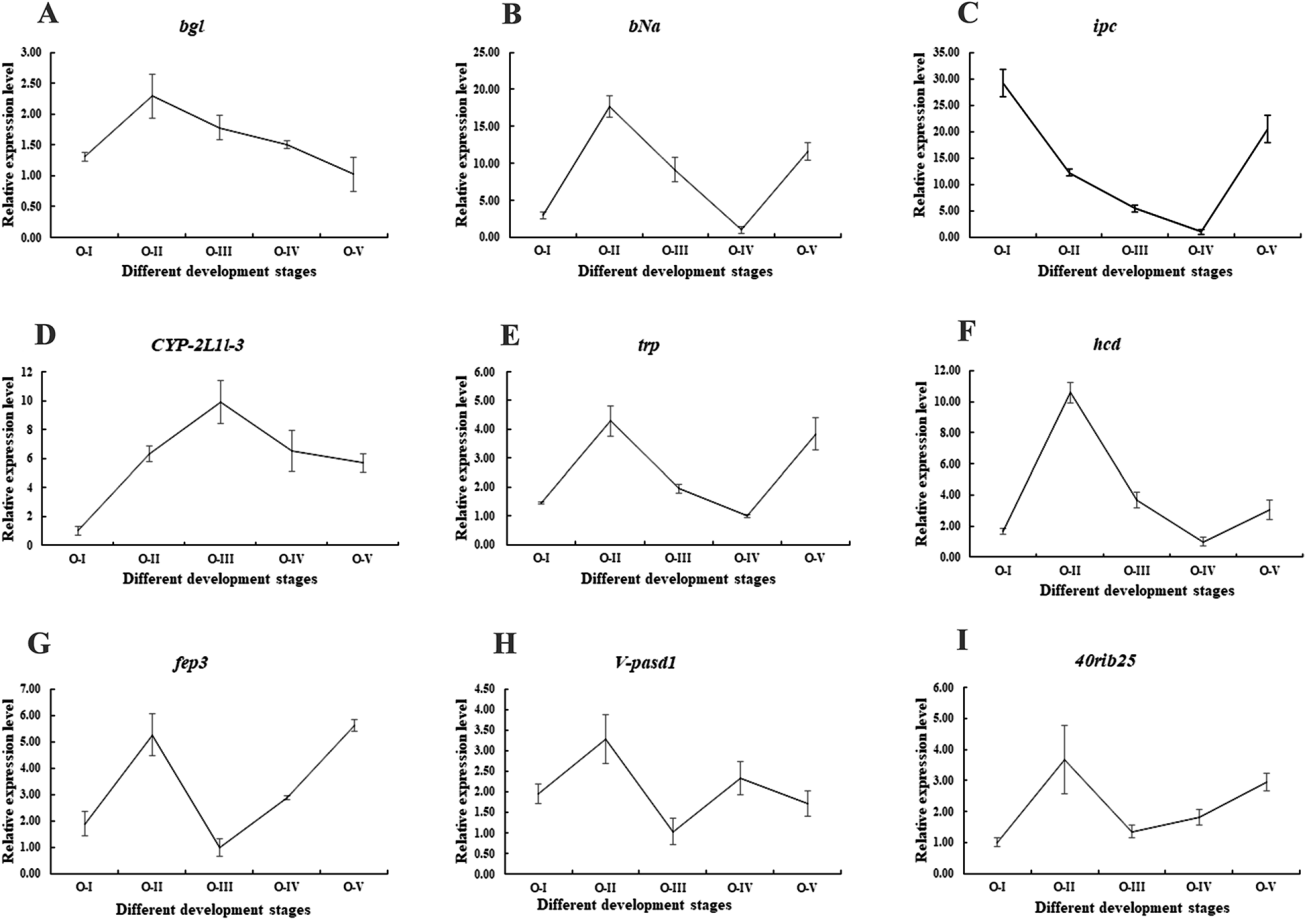
Expression profiles of 9 genes in five ovary stages. **A** *bgl*, **B** *bNa*, **C** *ipc*, **D** *CYP-2L1l-3*, **E** *trp*, **F** *hcd*, **G** *fep3*, **H** *V-pasd1*, I: *40rib25*. Different ovarian stages expressions: OI undeveloped stage, OII developing stage, OIII nearly-ripe stage, OIV ripe stage, OV spent stage. Data are shown as mean ± SD (*n* = 3). Statistical analyses were performed with one-way ANOVA analysis. Different letters denote significant differences (*P* < 0.05)

Furthermore, we analyzed the transcription levels of the nine genes during the five ovarian maturation stages (Fig. 5). *Bgl* showed the highest expression in O-II (*P* < 0.05), but was relatively low in other stages. The *bNa* also displayed significantly higher expression in O-II (*P* < 0.05), but very low expression in O-IV and O-I (*P* < 0.05). The *ipc* was negatively correlated with ovarian maturation (*P* < 0.05). *CYP-2L1l-3* showed dominant expression in O-III (*P* < 0.05), whereas *trp* had a similar expression profile to that of *bNa*. The *hcd* also showed its highest expression in O-II (*P* < 0.05), with relatively low expression in the other stages. *fep3* was expressed mainly expressed in O-II and O-V (*P* < 0.05). *V-pasd1* showed its highest expression in O-II (*P* < 0.05). *40rib25* displayed significant higher expression in O-II (*P* < 0.05), but was low levels in the other stages.

**Fig. 6:**
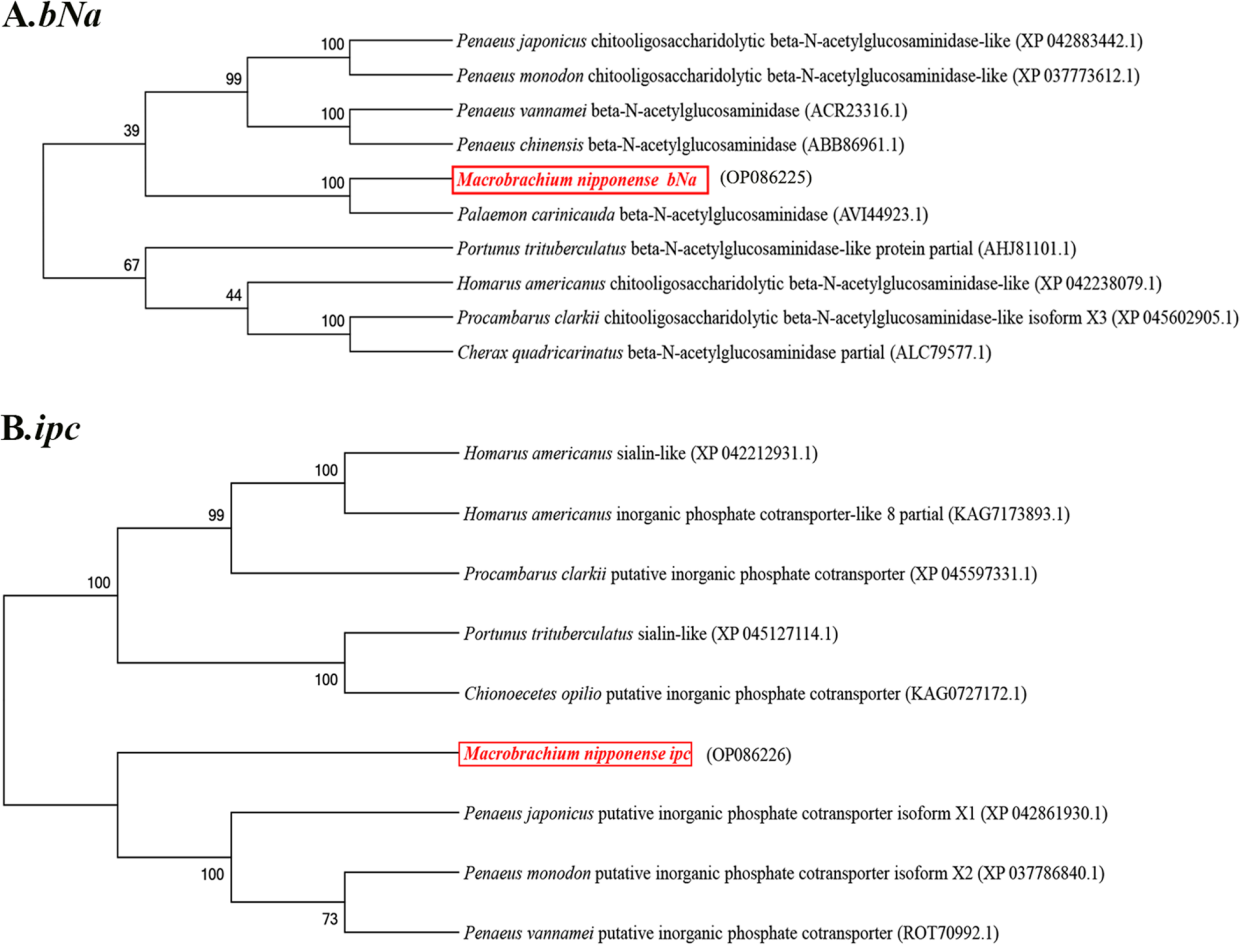
Phylogenetic tree of amino acid sequence. **A** *bNa,* **B** *ipc*. The numbers below the node indicate the bootstrap value

*The bNa* and *ipc* were selected to for evolutionary analysis. Several amino acid sequences of beta-*N*-acetylglucosaminidase and a putative inorganic phosphate cotransporter in crustaceans were deduced and a phylogenetic tree was built to determine the phylogenetic relationships based on the NJ method (Fig. 6A,B). The results showed that *bNa* of *M. nipponense* was most closely related to that from *Palaemon carincauda*. These clustered into one branch with other beta-*N*-acetyl glucosaminidases from *Penaeus japonicas*, *P. monodon*, *Penaeus vannamei*, and *Penaeus chinensis*. Beta-*N*-acetyl glucosaminidases from *Procambarus clarkii* and *Cherax quadricarinatus* were most closely related and clustered with *Homarus americanus* and *Portunus trituberculatus* into another branch. The putative inorganic phosphate cotransporter genes of *P. vannamei, P. monodon*, and *P. japonicas* first clustered together and then further clustered with *ipc* of *M. nipponense* into one branch. The genes from *P. clarkii*, *C. quadricarinatus*, and *H. americanus* first clustered together and then clustered with *P. trituberculatus* and *Chionoecetes opilio* into another branch. The gene from *P. chinensis* clustered alone into one branch.

## Discussion

The hepatopancreas is the largest digestive and nutrient storage organ in crustaceans and has an important role in carbohydrate and lipid metabolism, nutritional status, and energy storage and decomposition [24–26]. Nutrients are stored in the hepatopancreas and transported to gonads, muscles, and other tissues during growth and reproduction. It is also considered as a major lipid storage organ, similar to fat bodies in insects and vertebrates [27]. In crustaceans, the hepatopancreas is an important site for reproductive steroid hormone biosynthesis and catabolism and some biosynthetic steps in metabolic pathways, such as those producing sex steroid hormones and vitellogenin [26, 28–31]. Therefore, the hepatopancreas was hypothesized to have an important role in regulating gonad maturation in crustaceans. Previous studies of hepatopancreatic transcriptomes in *M. nipponense* mainly focused on hypoxic and nitrite stress, toxicity stress, virus infection, nutrition metabolism, and environmental stress [32–39]. In other crustaceans, hepatopancreas transcriptome analyses were performed to investigate the relationships between nutrition and male reproduction in *Eriocheir sinensis* [40]. To gain new insights into the role of the hepatopancreas in ovarian maturation of *M. nipponense*, we constructed systematic hepatopancreas transcriptomes from five ovary maturation stages in this species. In total, 90.46% of the clean reads obtained mapped to the *M. nipponense* reference genome [41], indicating that the transcriptomes had good coverage.

The GO enrichment results showed that DEGs was highly concentrated in synthesis and metabolic processes of nutrients and energy during ovarian maturation. Thus, the hepatopancreas was likely to undergo biological changes during ovarian development. During the HE-I–HE-III stages, the hepatopancreas, as the energy supply organ, was mainly involved in the synthesis and accumulation of energy substances, carbohydrates, and lipids. From HE-III to HE-IV, energy substrates were broken down and transported to the ovary. From HE-IV to HE-V, a new round of energy material synthesis began. KEGG enrichment results also indicated that nearly half of the pathways identified were involved in the synthesis, degradation, and metabolism of carbohydrates, lipids, and other nutrient intermediates, suggesting that the hepatopancreas has an important role in energy supply during ovarian maturation. In our previous ovary transcriptomes repots, the most significantly enriched pathway was the lysosome pathway (containing seven DEGs) in stage III versus IV, and this pathway was not significant enriched in the other comparison groups [6]. The gene encoding *cathepsin L* in this pathway shows higher expression in the hepatopancreas compared with ovary and has an important role in promoting ovarian maturation in *M. nipponense* [9, 13, 14]. Lysosomes were proposed to have an important part in the degradation of vitellogenin and were suggested to be involved in energy redistribution [42, 43]. Thus, it is widely accepted that lysosomes are related to gonadal development in most fish [14]. Crustacean ovary maturation involves the gradual accumulation of yolk, and the hepatopancreas provides nutrition for ovarian maturation [44, 45]. Carbohydrates, in the form of a variety of glycosylated products, are important functional groups in reproductive biology [46]. In zebrafish, the glycosaminoglycan biosynthesis pathway is associated with ovarian follicle activation [47]. In crustaceans, during ovarian growth, large amounts of glycoproteins are synthesized, forming the cortical vesicles and yolk granules of mature oocytes [48, 49]. In addition, lipids are also an important component of ovarian development [50]. In crustaceans, the most important energy materials transported to the ovary by the hepatopancreas is vitellogenin [51–53]. In decapods, as a precursor of Vitellin, vitellogenin is a high-density lipoprotein produced in the hepatopancreas in females and serves as an important source of proteins, lipids, and carbohydrates for the developing embryo [54]. The fatty acid degradation pathway is also associated with gonadal development in crabs [55, 56]. Based on the above research, we further hypothesized that the hepatopancreas of crustaceans regulated ovarian maturation through the lysosomal pathway.

We further analyzed the expression profiles of nine genes from these significantly enriched KEGG pathways in ovaries of different maturation stages. The results showed that seven DEGS were dominantly expressed in O-II, which is the vitellogenesis stage, indicating they might be involved in vitellogenesis. The above-described previous studies supported our results that enriched pathways from hepatopancreas were involved in ovary maturation. Furthermore, our results also suggested a new way to control rapid ovarian maturation in *M. nipponense* by regulating molecular nutritional signaling. As a next step, we will analyze the gene structure, spatial and temporal expression patterns, biological functions, and mutual regulatory relationships of these candidate genes in these important pathways to reveal their roles in ovarian maturation.

Conspicuously, “Insect hormone biosynthesis” was the only pathway enriched in the HE-II versus HE-III stage and participated in the development of the ovary from immaturity to maturity. This pathway is important in molting in insects. It mainly involves the synthesis of two terpenoids, ecdysteroid and juvenile hormone, which work in a synergistic manner to regulate development, metamorphosis, and reproduction [57–59]. In *M. nipponense*, as well as other crustaceans [60–62], females lay eggs after molting off the exoskeleton under favorable conditions, which means that the reproductive cycle occurs synchronously with the molting cycle. Many genes related to both ecdysteroid- and juvenile hormone signaling have been studied and proved to be involved in reproduction in crustaceans [63–66]. Thus, our results supported the important role of ecdysteroid and juvenile hormone signaling pathways in the hepatopancreatic regulation of ovarian maturation. In subsequent studies, we will conduct independent functional analysis of these DEG genes and determine their regulatory mechanisms in ovary maturation.

## Conclusions

Taken together, this study shed light on the role of the hepatopancreas in the molecular regulation of ovary maturation in *M. nipponense*. The results showed that nearly half of the significantly enriched pathways linked to nutrient metabolism and one insect hormone synthesis pathway were involved throughout ovarian maturation. This provided new insights into nutrition regulation and hormone regulation of ovarian maturation in crustaceans. Considerable efforts have been made to detect important pathways and functional genes related to the molecular nutritional-signaling regulation of reproduction. Subsequent functional validation studies of these candidate genes are necessary. We will analyze the gene structure, spatial and temporal expression patterns, biological functions, and mutual regulatory relationships of candidate genes in these important pathways to reveal their roles in ovarian maturation. Further studies on these genes and pathways will expand our understanding of the mechanism of fast ovary maturation in *M. nipponense*.

## Data Availability

All sequence reads were deposited in NCBI (accession SAMN27687877- SAMN27687891) under Bioproject PRJNA830321. The beta-*N*-acetylglucosaminidase and putative inorganic phosphate cotransporter genes of *M. nipponense* were deposited in NCBI (accession OP086225, OP086226). KEGG pathways were from Kanehisa Laboratories (https://www.kegg.jp/kegg/kegg1.html).

## References

[1] Kong Y, Ding Z, Zhang Y (2019). Types of carbohydrate in feed affect the growth performance, antioxidant capacity, immunity, and activity of digestive and carbohydrate metabolism enzymes in juvenile *Macrobrachium nipponense*. Aquaculture.

[2] Ministry of Agriculture Fisheries Bureau (2020). China fishery statistical yearbook.

[3] Jiang S, Xiong Y, Zhang W (2021). Molecular characterization of a novel Cathepsin L in *Macrobrachium nipponense* and its function in ovary maturation. Front Endocrinol.

[4] Qiao H, Fu H, Xiong Y (2017). Molecular insights into reproduction regulation of female oriental river prawns *Macrobrachium nipponense* through comparative transcriptomic analysis. Sci Rep.

[5] Wu P, Qi D, Chen L (2009). Gene discovery from an ovary cDNA library of oriental river prawn *Macrobrachium nipponense* by ESTs annotation. Comp Biochem Physiol Part D Genomics Proteomics.

[6] Zhang Y, Jiang S, Qiao H (2021). Transcriptome analysis of five ovarian stages reveals gonad maturation in female *Macrobrachium nipponense*. BMC Genomics.

[7] Jiang H, Li X, Sun Y (2016). Insights into sexual precocity of female oriental river prawn *Macrobrachium nipponense* through transcriptome analysis. PLoS One.

[8] Bai H, Qiao H, Li F (2015). Molecular characterization and developmental expression of vitellogenin in the oriental river prawn *Macrobrachium nipponense* and the effects of RNA interference and eyestalk ablation on ovarian maturation. Gene.

[9] Zhou Z, Fu H, Jin S (2021). Function analysis and molecular characterization of cyclin a in ovary development of oriental river prawn, *Macrobrachium nipponense*. Gene.

[10] Li F, Qiao H, Fu H (2018). Identification and characterization of opsin gene and its role in ovarian maturation in the oriental river prawn *Macrobrachium nipponense*. Comp Biochem Physiol B: Biochem Mol Biol.

[11] Bai H, Qiao H, Li F (2016). Molecular and functional characterization of the vitellogenin receptor in oriental river prawn, *Macrobrachium nipponense*. Comp Biochem Physiol A Mol Integr Physiol.

[12] Qiao H, Xiong Y, Zhang W (2015). Characterization, expression, and function analysis of gonad-inhibiting hormone in oriental river prawn, *Macrobrachium nipponense* and its induced expression by temperature. Comp Biochem Physiol A Mol Integr Physiol.

[13] Jiang H, Liu X, Li Y (2022). Identification of ribosomal protein L24 (RPL24) from the oriental river prawn, *Macrobrachium nipponense*, and its roles in ovarian development. Comp Biochem Physiol A Mol Integr Physiol.

[14] Zhu J, Fu H, Qiao H (2021). Expression and functional analysis of cathepsin L1 in ovarian development of the oriental river prawn, *Macrobrachium nipponense*. Aquacult Rep.

[15] Montes-Dominguez AL, Avena-Soto JA, Lizarraga-Rodriguez JL (2021). Comparison between cultured and wild Pacific white shrimp *(Penaeus vannamei)* vitellogenesis: next-generation sequencing and relative expression of genes directly and indirectly related to reproduction. PeerJ.

[16] Li K, Chen L, Zhou Z (2006). The site of vitellogenin synthesis in Chinese mitten-handed crab Eriocheir sinensis. Comp Biochem Physiol B: Biochem Mol Biol.

[17] Ding X, Nagaraju GPC, Novotney D (2010). Yolk protein expression in the green crab, *Carcinus maenas*. Aquaculture.

[18] Hiransuchalert R, Thamniemdee N, Khamnamtong B (2013). Expression profiles and localization of vitellogenin mRNA and protein during ovarian development of the giant tiger shrimp *Penaeus monodon*. Aquaculture.

[19] Jia X, Chen Y, Zou Z (2013). Characterization and expression profile of Vitellogenin gene from *Scylla paramamosain*. Gene.

[20] Trapnell C, Roberts A, Goff L (2012). Differential gene and transcript expression analysis of RNA-seq experiments with TopHat and cufflinks. Nat Protoc.

[21] Hu Y, Fu H, Qiao H (2018). Validation and evaluation of reference genes for quantitative real-time PCR in *macrobrachium Nipponense*. Int J Mol Sci.

[22] Yuan H, Zhang W, Fu Y (2021). MnFtz-f1 is required for molting and ovulation of the oriental river prawn *Macrobrachium nipponense*. Front Endocrinol.

[23] Livak KJ, Schmittgen TD (2001). Analysis of relative gene expression data using real-time quantitative PCR and the 2− ΔΔCT method. Methods.

[24] Vogt G (1994). Life-cycle and functional cytology of the hepatopancreatic cells of *Astacus astacus* (Crustacea, Decapoda). Zoomorphology.

[25] Wang L, Yan B, Liu N (2008). Effects of cadmium on glutathione synthesis in hepatopancreas of freshwater crab, *Sinopotamon yangtsekiense*. Chemosphere.

[26] Wang W, Wu X, Liu Z (2014). Insights into hepatopancreatic functions for nutrition metabolism and ovarian development in the crab *Portunus trituberculatus*: gene discovery in the comparative transcriptome of different hepatopancreas stages. PLoS One.

[27] Wen XB, Chen LQ, Ai CX (2001). Variation in lipid composition of Chinese mitten-handed crab, *Eriocheir sinensis* during ovarian maturation. Comp Biochem Physiol B: Biochem Mol Biol.

[28] Swevers L, Lambert JGD, De Loof A (1991). Metabolism of vertebrate-type steroids by tissues of three crustacean species comparative biochemistry and physiology part B: comparative. Biochemistry.

[29] Huang S, Wang J, Yue W (2015). Transcriptomic variation of hepatopancreas reveals the energy metabolism and biological processes associated with molting in Chinese mitten crab, *Eriocheir sinensis*. Sci Rep.

[30] Yang F, Xu HT, Dai ZM (2005). Molecular characterization and expression analysis of vitellogenin in the marine crab *Portunus trituberculatus*. Comp Biochem Physiol B: Biochem Mol Biol.

[31] Kung SY, Chan SM, Hui JHL (2004). Vitellogenesis in the sand shrimp, *Metapenaeus ensis*: the contribution from the hepatopancreas-specific vitellogenin gene (MeVg2). Biol Reprod.

[32] Sun S, Xuan F, Ge X (2014). Identification of differentially expressed genes in hepatopancreas of oriental river prawn, *Macrobrachium nipponense* exposed to environmental hypoxia. Gene.

[33] Xu Z, Li T, Li E (2016). Comparative transcriptome analysis reveals molecular strategies of oriental river prawn *Macrobrachium nipponense* in response to acute and chronic nitrite stress. Fish Shellfish Immunol.

[34] Zhao C, Fu H, Sun S (2018). A transcriptome study on *Macrobrachium nipponense* hepatopancreas experimentally challenged with white spot syndrome virus (WSSV). PLoS One.

[35] Gu X, Fu H, Sun S (2017). Dietary cholesterol-induced transcriptome differences in the intestine, hepatopancreas, and muscle of oriental river prawn *Macrobrachium nipponense*. Comp Biochem Physiol Part D Genomics Proteomics.

[36] Li F, Fu C, Xie Y (2019). Transcriptional responses to starvation stress in the hepatopancreas of oriental river prawn *Macrobrachium nipponense*. Environ Pollut.

[37] Zhu P, Wang H, Zeng Q (2021). Comparative transcriptome reveals the response of oriental river prawn (*Macrobrachium nipponense)* to sulfide toxicity at molecular level. Aquat Toxicol.

[38] Xu L, Fu Y, Fu H (2021). Transcriptome analysis of hepatopancreas from different living states oriental river prawn *(Macrobrachium nipponense*) in response to hypoxia. Comp Biochem Physiol Part D Genomics Proteomics.

[39] Yi C, Lv X, Chen D (2021). Transcriptome analysis of the *Macrobrachium nipponense* hepatopancreas provides insights into immunoregulation under Aeromonas veronii infection. Ecotoxicol Environ Saf.

[40] Jiang H, Yin Y, Zhang X (2009). Chasing relationships between nutrition and reproduction: a comparative transcriptome analysis of hepatopancreas and testis from *Eriocheir sinensis*. Comp Biochem Physiol Part D Genomics Proteomics.

[41] Jin S, Bian C, Jiang S (2021). A chromosome-level genome assembly of the oriental river prawn, *Macrobrachium nipponense*. GigaScience.

[42] Ayub Z, Ahmed M (2002). A description of the ovarian development stages of penaeid shrimps from the coast of Pakistan. Aquac Res.

[43] Zara FJ, Gaeta HH, Costa TM (2013). The ovarian cycle histochemistry and its relationship with hepatopancreas weight in the blue crab *Callinectes danae* (Crustacea: Portunidae). Acta Zool.

[44] Mourente G, Rodriguez A (1991). Variation in the lipid content of wild-caught females of the marine shrimp *Penaeus kerathurus* during sexual maturation. Mar Biol.

[45] Cavalli RO, Tamtin M, Lavens P (2001). Variations in lipid classes and fatty acid content in tissues of wild *Macrobrachium rosenbergii* (de man) females during maturation. Aquaculture.

[46] Dell A, Morris HR, Easton RL (1999). The glycobiology of gametes and fertilisation. Biochim Biophys Acta.

[47] Zhu B, Pardeshi L, Chen Y (2018). Transcriptomic analysis for differentially expressed genes in ovarian follicle activation in the zebrafish. Front Endocrinol.

[48] Kruevaisayawan H, Vanichviriyakit R, Weerachatyanukul W (2010). Oogenesis and formation of cortical rods in the black tiger shrimp, *Penaeus monodon*. Aquaculture.

[49] Pongtippatee-Taweepreda P, Chavadej J, Plodpai P (2004). Egg activation in the black tiger shrimp Penaeus monodon. Aquaculture.

[50] Wang H, Ding J, Ding S (2019). Metabolomic changes and polyunsaturated fatty acid biosynthesis during gonadal growth and development in the sea urchin *Strongylocentrotus intermedius*. Comp Biochem Physiol Part D Genomics Proteomics.

[51] Chen YN, Tseng DY, Ho PY (1999). Site of vitellogenin synthesis determined from a cDNA encoding a vitellogenin fragment in the freshwater giant prawn, *Macrobrachium rosenbergii*. Mol Reprod Dev.

[52] Tseng DY, Chen YN, Kou GH (2001). Hepatopancreas is the extraovarian site of vitellogenin synthesis in black tiger shrimp, *Penaeus monodon*. Comp Biochem Physiol A Mol Integr Physiol.

[53] Abdu U, Davis C, Khalaila I (2002). The vitellogenin cDNA of *Cherax quadricarinatus* encodes a lipoprotein with calcium binding ability, and its expression is induced following the removal of the androgenic gland in a sexually plastic system. Gen Comp Endocrinol.

[54] Khalaila I, Peter-Katalinic J, Tsang C (2004). Structural characterization of the N-glycan moiety and site of glycosylation in vitellogenin from the decapod crustacean *Cherax quadricarinatus*. Glycobiology.

[55] Yu ZB, Mu CK, Song WW (2015). Screening of genes related to ovarian development in the swimming crab, *Portunus trituberculatus*, by suppression subtractive hybridization. Genet Mol Res.

[56] Wang Q, Chen L, Wang Y (2012). Expression characteristics of two ubiquitin/ribosomal fusion protein genes in the developing testis, accessory gonad and ovary of Chinese mitten crab, *Eriocheir sinensis*. Mol Biol Rep.

[57] Spindler KD, Hönl C, Tremmel C (2009). Ecdysteroid hormone action. Cell Mol Life Sci.

[58] Riddiford LM (2012). How does juvenile hormone control insect metamorphosis and reproduction?. Gen Comp Endocrinol.

[59] Luo W, Liu S, Zhang W (2021). Juvenile hormone signaling promotes ovulation and maintains egg shape by inducing expression of extracellular matrix genes. Proc Natl Acad Sci.

[60] Sumiya E, Ogino Y, Miyakawa (2014). Roles of ecdysteroids for progression of reproductive cycle in the fresh water crustacean *Daphnia magna*. Front Zool.

[61] Gunamalai V, Kirubagaran R, Subramoniam T (2004). Hormonal coordination of molting and female reproduction by ecdysteroids in the mole crab *Emerita asiatica* (Milne Edwards). Gen Comp Endocrinol.

[62] Shyama SK (1987). Studies on moulting and reproduction in the prawn Macrobrachium idella (Heller). Mahasagar.

[63] Liu M, Xie X, Tao T (2016). Molecular characterization of methoprene-tolerant gene (met) in the swimming crab *Portunus trituberculatus*: its putative role in methyl farnesoate-mediated vitellogenin transcriptional activation. Anim Reprod Sci.

[64] Li X, Chen T, Han Y (2021). Potential role of Methoprene-tolerant (met) in methyl farnesoate-mediated vitellogenesis in the Chinese mitten crab (*Eriocheir sinensis*). Comp Biochem Physiol B: Biochem Mol Biol.

[65] Medesani DA, Ferré LE, Canosa IS (2015). Induction of vitellogenesis by 17-hydroxyprogesterone and methyl farnesoate during post-reproductive period, in the estuarine crab *Neohelice granulata*. Invertebr Reprod Dev.

[66] Miyakawa H, Sato T, Song Y (2018). Ecdysteroid and juvenile hormone biosynthesis, receptors and their signaling in the freshwater microcrustacean Daphnia. J Steroid Biochem Mol Biol.

